# Harnessing genomic resources for passion fruit improvement: Progress and prospects

**DOI:** 10.1002/tpg2.70213

**Published:** 2026-03-16

**Authors:** Khushboo Fulara, Vanika Garg, Xinhang Sun, Rebecca Ford, Natalie Dillon, Bruce Topp, Robert J. Henry, Mobashwer Alam, Rajeev K. Varshney

**Affiliations:** ^1^ WA State Agricultural Biotechnology Centre, Centre for Crop and Food Innovation, Food Futures Institute Murdoch University Murdoch Western Australia Australia; ^2^ Centre for Horticultural Science, Queensland Alliance for Agriculture and Food Innovation The University of Queensland Nambour Queensland Australia; ^3^ Centre for Planetary Health and Food Security Griffith University Nathan Queensland Australia; ^4^ Queensland Department of Primary Industries Mareeba Queensland Australia

## Abstract

Passion fruit (*Passiflora edulis*) is a highly nutritious horticultural crop cultivated widely across tropical and subtropical regions. Despite decades of breeding efforts that have led to the release of a few high‐yielding cultivars, on‐farm productivity remains suboptimal, and several existing cultivars are showing signs of declining vigor. To ensure the development of cultivars with stable and enhanced yields under both optimal and stress‐prone conditions, there is a growing impetus to improve breeding efficiency. Integrating advanced genomics technologies into conventional breeding pipelines offers a promising path forward. Over the past decade, substantial genomic resources have been developed, including genome‐wide markers, marker‐trait associations, reference genomes, and resequencing datasets. Some of these tools are already being deployed in breeding programs to enhance yield and consumer‐preferred traits. Emerging approaches such as genomic selection, speed breeding, and high‐throughput phenotyping hold further potential to accelerate genetic gains. Realizing the full benefits of these tools will require strategic utilization of diverse and targeted genetic resources, coupled with streamlined cultivar delivery systems. Addressing the technical and operational bottlenecks that hinder the translation of genomic advances to field‐ready cultivars will be key to securing the future of passion fruit improvement.

## Core Ideas

- Passion fruit breeding needs a boost to overcome low productivity and declining cultivar vigor.
- Genomics integration into breeding can improve yield, stress tolerance, and consumer‐preferred traits.
- Tools like genomic selection, speed breeding, and high‐throughput phenotyping can accelerate gains.
- Success depends on diverse genetic resources and efficient delivery of improved cultivars to farmers.

## Plain Language Summary

This review presents an update on the current status of passion fruit breeding, emphasizing the shift from traditional approaches to genomics‐assisted advancements. The paper emphasizes the constraints to passion fruit cultivation, such as loss of cultivar vigor, poor yields, and low tolerance to biotic and abiotic stresses. It suggests that modern genomics offers a promising path to enhance breeding efficiency and to overcome these limitations. Details of the progress made in developing genomic resources for *Passiflora* species, such as high‐quality reference genomes, molecular markers (e.g., single‐nucleotide polymorphisms and simple sequence repeats), and comprehensive transcriptomic datasets, are provided. These resources are foundational for understanding the genetic basis of key traits. The review discusses using functional omics and multi‐omics strategies to dissect the molecular basis of fruit quality, aroma, and stress response.

**Abbreviations**
AFLP: amplified fragment length polymorphism
AI: artificial intelligence
cDNA: complementary DNA
CRISPR: clustered regularly interspaced short palindromic repeats
DEG: differentially expressed genes
GS: genomic selection
GUS: β‐glucuronidase
GWAS: genome wide association studies
Hi‐C: high‐throughput chromosome conformation capture
IR: inverted repeat
LSC: large single‐copy
MAS: marker‐assisted selection
ML: machine learning
*NBS‐LRR*
: nucleotide‐binding site leucine rich repeat
PAVs: presence/absence variations
PGD: passion fruit genomic database
qRT‐PCR: quantitative real‐time PCR
QTL: quantitative trait locus
RAPD: random amplified polymorphic DNA
RNA‐seq: RNA sequencing
rRNA: ribosomal RNA
SMRT: single‐molecule real‐time sequencing
SNP: single‐nucleotide polymorphism
SSC: small single‐copy
SSR: simple sequence repeat
SVs: structural variations
T2T: telomere to telomere
tRNA: transfer RNA
VOCs: volatile organic compounds
WGD: whole‐genome duplication
XAP: *Xanthomonas axonopodis* pv. *passiflorae*

## 1 INTRODUCTION

Passion fruit (*Passiflora edulis*) is a nutrient‐rich fruit valued for its high fiber content, essential vitamins (A and C), minerals, and organic acids (Ramaiya et al., 2012, 2019). Its diverse applications in the food industry, livestock feed, and traditional medicine, coupled with its adaptability to diverse climatic conditions, contribute to its increasing popularity in global markets (Choi, 2025; Ferreira et al., 2021). This globally valued fruit belongs to the *Passifloraceae* family, which is dominated by the genus *Passiflora*, a species‐rich and commercially significant clade of plants comprising approximately 600 species worldwide, most of which are lianas. The large diversity within *Passiflora* necessitates a complex taxonomic classification into four subgenera: *Astrophaea, Decaloba, Deidamiodes*, and the largest, *Passiflora*. This infrageneric classification is supported by morphological, phylogenetic, and cytogenetic evidence, including different basic chromosome numbers (*x* = 6, 9, 10, 11, or 12) associated with each subgenus (Cerqueira‐Silva et al., 2014; Xia et al., 2021).

Global production of passion fruit exceeds 1.4 million metric tons (mmt) annually, cultivated across an estimated 14.5 million ha, with a market value of approximately USD 400 million (Khuwijitjaru & Klinchongkon, 2020). South America dominates production, contributing 65% of global output, while Australia and New Zealand account for 3% and 2.2%, respectively. The major producers are Brazil (0.6 mmt), Ecuador (0.13 mmt), Indonesia (0.1 mmt), India (0.07 mmt), and Colombia (0.05 mmt; FAOSTAT, 2024). The subgenus *Passiflora* alone encompasses approximately 240 species. Of these, about 50 species yield edible fruits, primarily originating from the outer fringes of South American rainforests, particularly the Amazon region of Brazil, Paraguay, and northern Argentina. Key edible species include *P. edulis, P. quadrangularis, P. ligularis, P. tripartita* var. *mollissima, P. tarminiana*, and wild relatives such as *P. incarnata*. However, the exact origin of the yellow passion fruit remains uncertain, with competing theories suggesting Brazil, Australia, or elsewhere in South America as its possible place of emergence (Akamine & Girolami, 1959; Seale & Sherman, 1960).

While many *Passiflora* species are consumed locally, their high perishability has largely limited their success in export markets (Castillo et al., 2021). In contrast, *P. edulis* is the primary cultivated species worldwide, and most current research focuses on this species (Z. C. Pereira et al., 2023). *P. edulis* is a semi‐perennial tropical vine, utilizing tendrils to climb, with stems reaching up to 15 m in length. Flowers of these species are hermaphroditic and protandrous, which, combined with sticky pollen, makes them self‐incompatible, thus requiring cross‐pollination for optimal fruit set and yield. Despite the importance of *P. edulis* as a fruit crop, long‐term breeding programs are still in the early stages and face considerable challenges such as existing cultivars experiencing declining vigor, possibly due to a narrow genetic base, inability to adapt to changing climatic conditions, chronic infection by insects and disease, and natural senescence due to a short productive life (Nichols, 2023). Moreover, due to the semi‐perennial nature of the vines, growers have to conduct repetitive clonal propagation, which may contribute to yield decline. On the other hand, outcrossing and self‐incompatibility in *P. edulis* shape its genetic structure, maintaining extensive allelic diversity while limiting the development of uniform, true‐to‐type cultivars. A clear understanding of its reproductive genetics is thus essential to overcome these biological barriers, optimize breeding strategies, design effective crossing schemes, and facilitate genomic‐assisted breeding.

Other *Passiflora* species, such as *P. alata*, are gaining interest for their ornamental appeal and exotic‐flavored fruits; however, no commercial varieties are currently available to farmers (Z. P. D. Costa et al., 2017). Similarly, the biodiversity inherent in wild passion fruit species, such as *P. cincinnata, P. nitida, P. incarnata*, and *P. setacea*, along with their resistance to abiotic and biotic stress factors, represents a valuable resource for breeding programs, although research in these areas remains in its early stages. Table 1 summarizes the *Passiflora* species currently utilized as fruiting parents or as sources of cold and disease tolerance in breeding programs.

**TABLE 1:** Key *Passiflora* species utilized as fruiting parents or as sources of cold and disease tolerance in breeding programs.

Species	Common name	Origin	Description of fruit	Environmental adaptation	Areas of cultivation or importance	References
*P. quadrangularis*	Giant granadilla	Peru/Brazil	Very large fruit (20–30 cm long), green‐yellow skin, oblong shape, white plink flesh with central cavity filled with purple/pink delicately flavored pulp	Warm, wet tropical lowland (>13°C)	Cultivated in all tropical regions of the world, including Northern Queensland	Rojas‐Sandoval (2022)
*P. ligularis*	Sweet granadilla	Venezuela/Brazil	Large fruit (7–8 cm diameter), orange/brown to yellow/tan when ripe, hard shell, ovoid shape, white edible aromatic pulp	Tropical highland (1000‐3000 m)	Cultivated in Mexico, California, and Central Tropical America	Lim (2012)
*P. mollissima*	Banana passion fruit	Andes of tropical South America	Large, ellipsoidal fruit (7 cm long), yellow skin with orange/salmon colored aromatic and edible pulp	Suited to 2000–3000 m in the tropics	Cultivated in East Columbia, Southeast Peru, West Bolivia, and West Venezuela	Popay (2012)
*P. incarnata*	Vine apricot or wild passion fruit	Subtropical and tropical North America/Bermuda	Medium‐sized, ovoid‐shaped fruit (5 cm diameter), yellow skin and pulp	Suited to warm subtropics, strong perennial vine, roots survive frost	Cultivated as a garden specimen in the Southern United States	Bokelmann (2021)
*P. caerulea*	Blue passion fruit	Brazil	Medium‐sized, ovate‐ to sub‐globose‐shaped fruit (5 cm long), yellow‐skinned	Suited to cooler tropics and subtropics	Used as a rootstock in South Africa	Lusweti et al. (2011)
*P. alata*	Winged stem passionflower	Peru/Brazil	Large‐sized, obovoid‐shaped fruit (8‐10 cm long), thin yellow skin	Tropical lowland	Cultivated widely in subtropical and tropical areas of the world	Taiwe and Kuete (2017)
*P. coccinea*	Red passionflower, red granadilla	Northeastern and Southern America	Medium‐sized, ovoid‐shaped fruit (5 cm long), striped, brittle, and mottled yellow skin	Tropical regions	Occasionally cultivated in some areas of the world	Yockteng et al. (2011)

One way to introgress beneficial traits in *P. edulis* is through interspecific hybridization. However, this technique also poses some inherent challenges. Although such crosses in *Passiflora* are manageable, their success is highly dependent on the species involved. This is due to several pre‐zygotic and post‐zygotic barriers, including pollination success, hybrid lethality resulting from plastome‐genome incompatibility, and other factors such as unilateral incompatibility (Bugallo et al., 2025; Mráček, 2005; Soares et al., 2020). Moreover, the genetic resources available for breeding improved varieties with superior agronomic traits, high fruit quality, and resistance to pests and diseases remain limited. To date, no single genotype has successfully combined all these desirable traits, making genetic improvement a major bottleneck for sustainable passion fruit production.

Recent advances in genomics and molecular breeding have provided new opportunities to enhance passion fruit improvement (Savadi et al., 2021). The availability of high‐throughput DNA markers, transcriptome assemblies, and whole‐genome sequencing data are beginning to unravel the genetic basis of key agronomic traits. While these genomic resources remain largely restricted to a few *Passiflora* species, integrating modern breeding approaches such as genomic selection (GS) and rapid generation turnover offers promising avenues to accelerate genetic gains. The application of these cutting‐edge techniques, coupled with the strategic exploration of genetic diversity, has the potential to transform passion fruit breeding and ensure long‐term sustainability.

This review synthesizes recent progress in *Passiflora* genomics, highlights the development of next‐generation genetic resources and molecular tools, and explores their integration into breeding pipelines. We also discuss strategies for harnessing genetic diversity and applying modern breeding methodologies to enhance the productivity, resilience, and marketability of passion fruit across diverse agroecological zones.

**Core Ideas**

- Passion fruit breeding needs a boost to overcome low productivity and declining cultivar vigor.
- Genomics integration into breeding can improve yield, stress tolerance, and consumer‐preferred traits.
- Tools like genomic selection, speed breeding, and high‐throughput phenotyping can accelerate gains.
- Success depends on diverse genetic resources and efficient delivery of improved cultivars to farmers.

## 2 GENETIC RESOURCES FOR TRAIT DISCOVERY AND UTILIZATION

The diversity of crop genetic resources is fundamental to fulfilling global food and nutritional demands, while also representing an important asset for plant breeding and crop improvement. Various *Passiflora* species are cultivated across the tropical and subtropical regions of the world. Historical studies suggest that the “naturalization” of passion fruit occurred in South Africa, Hawaii, California, Florida, Kenya, and Sri Lanka by the end of the 19th century, and in Fiji by the middle of the 20th century. Brazil and Colombia are widely recognized as the primary centers of *Passiflora* diversity, hosting around 30% of known species, approximately 150 in Brazil and 170 in Colombia, including 89 that are endemic to Brazil (Fajardo et al., 1998; Ocampo et al., 2010; Viana et al., 2003). This rich germplasm reservoir, conserved across multiple international genebanks, holds immense potential for accelerating trait discovery and innovation in passion fruit breeding. Globally, >50 collections representing at least 1200 accessions are maintained across 32 countries. Of these, approximately 95% of accessions are conserved by germplasm banks in nine countries: Brazil (32%), Ecuador (30%), Peru (14%), Colombia (8%), Australia (5%), France (3%), United States (2%), Costa Rica (2%), Jamaica (2%), and Kenya (2%) (Cerqueira‐Silva et al., 2015; Sun et al., 2025). The germplasm collections of Brazil, Kenya, Colombia, and Australia have been instrumental in investigating the genetic diversity and population structure among various passion fruit accessions (Cerqueira‐Silva et al., 2014; Oluoch et al., 2018; Rodríguez‐Castillo et al., 2021; Sun et al., 2025). Similarly, the Ecuadorian germplasm has facilitated studies on important agronomic traits, including plant yield, fruit quality, pulp mineral content, phytochemical composition, and antioxidant activity (Viera et al., 2020; Viera, Shinohara, Samaniego, Sanada, et al., 2022; Viera, Shinohara, Samaniego, Terada, et al., 2022). Several additional national and international *Passiflora* germplasm collections are maintained by institutions worldwide, including the Embrapa Cassava and Fruits research unit (Embrapa Mandioca e Fruticultura, Bahia, Brazil), the Biology Department of Universidad Nacional de Colombia (Bogotá, Colombia), La Selva Experimental Station, Universidade Estadual do Norte Fluminense Darcy Ribeiro (Rio de Janeiro, Brazil), Mississippi State University (Mississippi, USA), and the University of Florida's Tropical Research and Education Center (Florida, USA; Rionegro, Antioquia; Anderson et al., 2022; J. L. Costa et al., 2012; Rodríguez‐Castillo, Ambachew, et al., 2020; Rodríguez‐Castillo, Melgarejo, et al., 2020). These germplasm collections preserve a wide variety of commercial cultivars and landraces, some of which have been instrumental in facilitating our understanding of structural variability of the seed, germplasm capacity, and photosynthetic and physiological adaptations under contrasting environmental conditions (Rodríguez‐Castillo et al., 2019; Rodríguez‐Castillo, Melgarejo, et al., 2020).

## 3 ORPHAN NOT ANYMORE: GENOMIC RESOURCES

**FIGURE 1:**
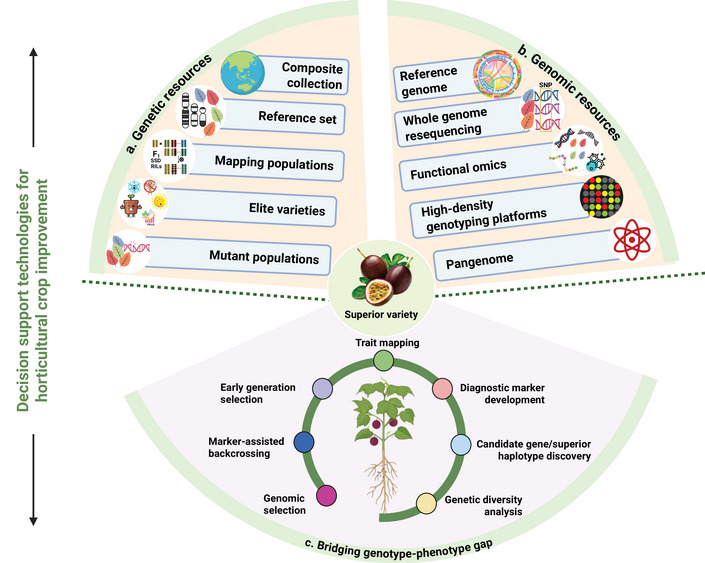
Applications of genomic technologies for bridging the genotype‐phenotype gap in passion fruit. The figure provides an overview of integrating genetic resources together with genomic resources and technologies for bridging the genome‐to‐phenome gap to produce improved passion fruit varieties. Germplasm resources for passion fruit, such as composite collections, mini‐core, and reference sets, can be subjected to sequencing/genotyping. A similar approach can be followed for elite varieties, mapping populations segregating for important agronomic traits, and mutant populations. Once the reference genome becomes available, these genetic resources can be subjected to whole‐genome re‐sequencing or high‐to‐low‐density genotyping, based on the objective of the study, using the available genotyping platforms (e.g., genotyping by sequencing, array‐based genotyping). Subsequently, analyses at the transcriptome, proteome, and metabolome levels can be performed to gain novel insights into the candidate genes and biological processes involved. A pangenome can also be constructed to capture the entire set of genes from *Passiflora* species. Analysis of this sequencing/genotyping data, along with phenotyping data with high‐throughput decision support technologies, can provide solutions for genetic diversity analysis, genetic mapping, and quantitative trait locus (QTL) analysis; identify candidate genes and superior haplotypes; and develop diagnostic markers, early generation selection, marker‐assisted backcrossing, and genomic selection (GS). Integration of such resources should bridge the genotype‐phenotype gap and accelerate the development of climate change‐ready varieties with higher yields, improved resistance against biotic and abiotic stresses, and enhanced genetic gains in farmers’ fields.

Owing to the historical scarcity of genetic and genomic resources, passion fruit was long considered an under‐researched “orphan crop” (Rodriguez‐Castillo et al., 2021). However, since 2021, rapid advances in next‐generation sequencing technologies have led to the development of diverse genetic, genomic, and transcriptomic resources, effectively transforming passion fruit from an orphan crop into a genomic resource‐rich crop (Carneiro et al., 2002; Z. P. Costa et al., 2021; Cutri & Dornelas, 2012; Ma et al., 2021; G. S. Pereira et al., 2013; Xia et al., 2021; Zheng et al., 2024). The availability of chromosome‐scale draft genome assemblies, large‐scale resequencing datasets, molecular markers, and low‐ to high‐density genotyping assays has enabled the application of translational genomics in passion fruit breeding (Castillo et al., 2021; Z. P. Costa et al., 2021; Ma et al., 2021; A. A. Santos et al., 2014; Wu et al., 2025; Xia et al., 2021; Yu et al., 2024; Zheng et al., 2024). These resources are enabling more targeted approaches to trait dissection and marker‐assisted selection (MAS). The development of ultra‐high‐throughput and cost‐effective genotyping platforms will be essential for scaling the use of genomics in practical breeding programs. Such platforms will support large population studies, GS, and the efficient identification of favorable alleles for complex traits. Figure 1 highlights the importance of genomic technologies and resources available in passion fruit for bridging the genotype‐phenotype gap.

### 3.1 Molecular markers for genetic analysis

The advent of high‐throughput sequencing technologies has transformed crop genomics by enabling the generation of large‐scale genomic datasets and facilitating cost‐effective, high‐resolution genotyping (Rasheed et al., 2017). Molecular markers are indispensable tools in modern plant breeding, offering powerful means to assess genetic diversity, construct linkage maps, and identify loci associated with agronomically important traits. In passion fruit, the availability of molecular markers has progressed significantly over the past two decades, laying a critical foundation for genomics‐assisted breeding.

Initial studies on *Passiflora* genetics employed first‐generation molecular markers such as restriction fragment length polymorphism (Sánchez et al., 1999), random amplified polymorphic DNA (RAPD), and amplified fragment length polymorphism (AFLP) to assess genetic variation and conduct preliminary trait mapping. These techniques, while pioneering, were limited by low reproducibility and scalability. The development of simple sequence repeat (SSR) markers marked a major advance in passion fruit genetics, offering co‐dominant inheritance, high polymorphism, genome‐wide distribution, and ease of use. Initially, developing SSR markers from genomic libraries or expressed sequence tags using Sanger sequencing was expensive and time‐intensive. However, SSRs remained the marker of choice in many crops from 2000 to 2010 due to their reliability and ease of genotyping (Deschamps et al., 2012).

Recent efforts have significantly increased SSR resources in passion fruit. High‐throughput SSR identification of the *Passiflora* genome revealed 13,104 perfect SSRs, predominantly comprising di‐ to tetranucleotide repeats (Wu et al., 2018). In Kenya, SSR‐based genetic analysis of 50 purple passion fruit genotypes uncovered significant genetic variability, offering valuable insights for germplasm conservation and breeding (Chepkoech et al., 2020). Additionally, SSR markers have been developed for genes associated with disease resistance, particularly the *nucleotide‐binding site leucine‐rich repeat* (*NBS‐LRR*) gene family. A recent study identified 497 microsatellite loci in 161 *NBS‐LRR* genes, with 25 polymorphic SSR markers validated for genetic studies (Trung et al., 2024). These SSR‐based resources provide crucial tools for germplasm evaluation, genetic improvement, and molecular breeding in passion fruit.

In recent years, single‐nucleotide polymorphisms (SNPs) have emerged as the preferred choice of markers due to their high abundance, genome‐wide distribution, and compatibility with next‐generation sequencing platforms (Deschamps et al., 2012). SNPs and SSRs have been characterized in both *P. edulis* and *P. alata*, with an estimated SNP frequency of one per 294 bp in *P. alata* (Z. P. D. Costa et al., 2017). Genotyping‐by‐sequencing approaches have uncovered greater genetic diversity in purple passion fruit landraces compared to commercial cultivars, underscoring the importance of conserving semi‐wild and traditional varieties as reservoirs of useful alleles (Castillo et al., 2021).

Additionally, restriction site‐associated DNA sequencing has been employed across multiple *Passiflora* species, elucidating phylogenetic relationships and facilitating the development of 2515 SSR primer pairs. Notably, cultivated passion fruit exhibited the highest SSR transferability across species, supporting its utility in comparative genomics and MAS (Wu et al., 2020). These advancements have strengthened the genetic foundation for passion fruit improvement programs. Further, the integration of small cost‐effective SNP panels for early‐generation screening, parent verification, and identification of true F1 hybrids is expected to become more prevalent in passion fruit breeding programs. These cost‐effective genotyping strategies will accelerate genetic gains and contribute to the development of improved, high‐performing cultivars.

### 3.2 Genetic mapping and identification of quantitative trait loci (QTLs) for agronomically important traits

Genetic mapping provides a foundational framework for identifying chromosomal regions linked to agronomically important traits and is an essential prerequisite for modern molecular breeding. These maps complement sequencing efforts by providing information on recombination frequencies, marker order, and trait‐marker associations across the genome. In recent years, significant progress has been made in genetic mapping studies in passion fruit. The first genetic linkage map for passion fruit was developed using biparental populations derived from a cross between two clones of yellow passion fruit, IAPAR123 (female) and IAPAR06 (male), using 380 RAPD markers. The linkage map of IAPAR123 consisted of 135 markers distributed across nine linkage groups, spanning a total length of 727.7 centimorgan (cM), with an average marker distance of 11.2 cM. Similarly, the linkage map of IAPAR06 comprised 96 markers, spanning 783.5 cM with an average distance of 12.2 cM. On average, these parental maps provided approximately 61% genome coverage (Carneiro et al., 2002). A subsequent study using the same varieties utilized 174 AFLP markers to develop updated linkage maps. The linkage map of IAPAR123 consisted of 140 markers mapped with a marker density of 9.6 markers per cM, covering 488.9 cM. For IAPAR06, the map included 115 markers mapped with a marker density of 12.6 per cM, covering 790.2 cM (Lopes et al., 2006). Since passion fruit is self‐incompatible, the maps were constructed using a “two‐way pseudo‐testcross” strategy based on monoparental dominant markers segregating in a 1:1 ratio. As a result, individual parental maps were generated, lacking marker information from the non‐segregating parent. Later, an integrated molecular map was reconstructed for a full‐sib population of yellow passion fruit using biparental markers. This genetic map was constructed using 253 AFLP markers and 107 SSR markers, resulting in a total of ten linkage groups and covering 1687 cM. On average, 24 markers were assigned per linkage group, with a marker density of 6.9 cM (Oliveira et al., 2008).

For the sweet passion fruit species, *P. alata*, a full‐sib progeny (derived from a single cross between female parent “2(12)” and male parent “SV3”) was used to estimate genetic parameters and map QTLs for seven fruit traits. The traits evaluated included fruit diameter, length, weight, thickness of fruit skin, weight of fruit skin, weight of fruit pulp, and soluble solids. The analysis identified 22 QTLs mapped in seven of the nine linkage groups, with individual trait heritabilities ranging from 59.8% to 82.7%. The proportion of phenotypic variance explained by each QTL ranged from 42% to 64.3% (G. d. S. Pereira et al., 2017). These genetic mapping efforts provided valuable tools for passion fruit breeding programs, enabling the identification of genomic regions associated with important agronomic traits. However, the aforementioned genetic maps suffered from low marker density, limited genome coverage, and low detection efficiency, thus limiting their application. To overcome these limitations, a high‐density linkage map was recently constructed by resequencing a BC_1_F_1_ population from *P. edulis*. The linkage map was constructed with 1,180,406 SNPs distributed across nine linkage groups, spanning a total length of 1559.03 cM. The average genetic distance per linkage group was 311.81 cM. One QTL, *qPSR5*, was identified for passion fruit stem rot resistance, located between 145.878–152.951 cM on chromosome 5, explaining 8.6% of phenotypic variance (Wu et al., 2025). Together, these genetic and QTL mapping efforts have established a critical platform for dissecting complex traits in passion fruit and accelerating the development of improved cultivars through molecular breeding.

### 3.3 Reference genomes to delineate genomic architecture

A high‐quality reference genome is a foundational resource for unraveling genome structure, identifying trait‐associated loci, and tracing evolutionary and domestication events in crops (Garg et al., 2022, 2024). In passion fruit, recent advances in sequencing technologies and assembly strategies have enabled the construction of increasingly complete and accurate reference organellar and nuclear genomes, thereby providing a critical platform for trait discovery and molecular breeding.

### 3.4 Organellar genomes

Mitochondria and chloroplasts are specialized organelles derived from ancient endosymbiosis in eukaryotic cells, each maintaining its own genome essential for respiration and photosynthesis, respectively (Arimura & Nakazato, 2024). These organellar genomes exhibit distinct characteristics, including multiple copies per cell, predominantly maternal inheritance, unique genomic structures, and frequent homologous recombination. Sequencing and characterizing organellar genomes offer valuable insights into plant evolutionary history, cytoplasmic inheritance, and stress adaptation mechanisms.

To date, a mitochondrial genome assembly has been assembled for yellow passion fruit. This genome, measuring 0.68 Mb, was sequenced using the Illumina HiSeq 2000 platform. A total of 74 genes were identified, including 41 protein‐coding genes, three ribosomal RNA (rRNA) genes, and 30 transfer RNA (tRNA) genes. The protein‐coding regions comprised 64.7% of the genome (440,290 bp), while tRNA and rRNA genes accounted for 2218 bp and 5454 bp, respectively (Cauz‐Santos et al., 2017).

In contrast, the chloroplast genome of yellow passion fruit has been extensively studied. The first complete chloroplast genome for the yellow passion fruit variety IAPAR123 was assembled using PacBio data of two bacterial artificial chromosome clones (*Pe69Q4G9* and *Pe85Q4F4*; L. A. C. Santos et al., 2017). This 151 kb genome features two inverted repeat (IR) regions (IRA and IRB) of 26,154 bp each, flanking a small single‐copy (SSC) region of 13,378 bp and a large single‐copy (LSC) region of 85,720 bp. Annotation revealed 105 unique genes, including 71 protein‐coding genes, 30 tRNAs, and four rRNAs, along with 36 repetitive elements and 85 SSRs. Notably, three structural rearrangements (inversions of 46,151 bp, 3765 bp, and 1631 bp) were detected in the LSC region, distinguishing this chloroplast genome from those of related species.

A second complete chloroplast genome was assembled for the purple passion fruit cultivar Pintang No. 1 using the BGISEQ‐500 platform (Yang et al., 2019). This 152 kb genome contains a pair of IR regions (25,989 bp each), an SSC region (13,352 bp), and an LSC region (85,141 bp). A total of 111 unique genes were annotated, including 77 protein‐coding genes, 30 tRNAs, and four rRNAs.

Additionally, complete chloroplast genomes have been reported for several other *Passiflora* species and interspecific hybrids, including an artificial hybrid (*P. edulis* × *P. edulis* f. *edulis*), *P. caerulea*, and most recently, *P. tripartita* var. *mollissima*. A detailed overview of these assemblies is provided in Table 2.

**TABLE 2:** Detailed overview of passion fruit chloroplast genome assemblies developed to date.

							Predicted genes				
Species	Genotype	Assembly size (Kb)	Sequencing technology used	IR (bp)	SSC (bp)	LSC (bp)	Unique	Protein coding	Transfer RNA	Ribosomal RNA	References
*P. edulis* f. *flavicarpa*	IAPAR123	151	PacBio RS II	26,154	13,378	85,720	105	71	30	4	Cauz‐Santos et al. (2017)
*P. edulis*	Pintang no. 1	152	BGI seq‐500	25,989	13,352	85,141	111	77	30	4	Yang et al. (2019)
Artificial hybrid (*P. edulis* × *P. edulis* f. *edulis*)	Ziyan	151	Illumina Hiseq 2000	26,155	13,378	86,628	131	86	37	8	Feng et al. (2020)
18 Species from *Passiflora* (*P. cerradensis*, *P. haematostigma*, *P. rhamnifolia*, *P. candollei*, *P. capsularis*, *P. costaricensis*, *P. suberosa*, *P. vespertilio*, *P. contracta*, *P. deidamioides*, *P. alata*, *P. cristalina*, *P. edmundoi*, *P. loefgrenii*, *P. miniata*, *P. mucronata*, *P. recurva*, *P. watsoniana*)	–	148 on average	Illumina NextSeq	431,215 on average	11,882 on average	73,464 on average	104 on average	70 on average	30	4	Cauz‐Santos et al. (2020)
*P. caerulea*	–	151	Illumina Hiseq 2500	26,180	13,397	85,623	131	79	37	8	Niu et al. (2021)
*P. tripartita* var. *mollissima*	–	163	Illumina NovaSeq 6000	32,204	13,518	85,525	110	84	36	8	Aliaga et al. (2024)

Abbreviations: IR, inverted repeat; LSC, large single‐copy; SSC, small single copy.

### 3.5 High‐quality nuclear genomes

In parallel with efforts to characterize organellar genomes, significant strides have been made in high‐quality nuclear genome assembly of passion fruit. In the past, six genome assemblies of *Passiflora* spp. have been sequenced (Table 3), providing foundational resources for gene discovery, structural analysis, and breeding applications. The first draft nuclear genome assembly of *P. edulis* was generated using Oxford Nanopore Technology long‐read sequencing, combined with high‐throughput chromosome conformation capture (Hi‐C) scaffolding. The resulting 1341.7 Mb genome had a scaffold N50 of 3.1 Mb, with 98.92% of the sequence anchored to nine chromosomes. Annotation predicted 23,171 protein‐coding genes, with a large portion of the genome composed of repetitive elements, primarily long terminal repeat retrotransposons concentrated near centromeres. This assembly offered initial insights into genes associated with fruit flavor biosynthesis and chromosomal organization (Xia et al., 2021). A second genome assembly of *P. edulis* was later produced using PacBio high‐fidelity long reads and Hi‐C scaffolding. This assembly, totaling 1270 Mb, predicted 39,309 protein‐coding genes and revealed 56 basic leucine zipper transcription factors associated with hormone response and stress signaling pathways, including abscisic acid, drought, and light signaling. The improved contiguity and annotation offered enhanced resolution for studying regulatory networks and stress resilience (Ma et al., 2021).

**TABLE 3:** An overview of passion fruit assemblies.

Species	Genotype	Ploidy	Estimated genome size (Mb)	Platform	Assembly size (Mb)	N50 contig (Mb)	N50 scaffold (Mb)	BUSCO (%)	Predicted genes	References
*P. edulis* f. *flavicarpa*	IAPAR‐123	2*n* = 2*x* = 18	1395.76	ABI 3500 × L	–	–	–	–	∼900 BAC‐end sequences	A. A. Santos et al. (2014)
*P. edulis*	Purple passion fruit	2*n* = 2*x* = 18	1395.76	ONT, Illumina Novaseq 6000, Hi‐C	1341.7	6.4	3.1	91.56	23,171	Xia et al. (2021)
*P. edulis*	Purple passion fruit	2*n* = 2*x* = 18	1395.76	PacBio Sequel II, Illumina HiSeq × Ten, Hi‐C	1270	0.7	126.4	99.22	39,309	Ma et al. (2021)
*P. organensis*	AF82F07 (National Genetic Heritage and Associated Traditional Knowledge Management System, Brazil)	2*n* = 2*x* = 6	259	PacBio SMRT, IlluminaHiSeq	259.3	2.4	8.2	98.4	25,327	Z. P. Costa et al. (2021)
*P. edulis*	Zihua	2*n* = 2*x* = 18	1395.76	PacBio SMRT, IlluminaHiSeq, Hi‐C	1375.4	9.82	–	98.6	30,670	Zheng et al. (2024)
*P. edulis* f. *flavicarpa*	Shaohuang	2*n* = 2*x* = 18	1395.76	PacBio SMRT, IlluminaHiSeq, Hi‐C	1357.5	43.9	–	99.3	29,425	Zheng et al. (2024)

Abbreviations: Hi‐C, high‐throughput chromosome conformation capture; ONT, Oxford Nanopore Technology; SMRT, single‐molecule real‐time sequencing.

Most recently, chromosome‐scale genomes for *P. edulis* and *P. edulis* f. *flavicarpa* were assembled using a combination of PacBio Sequel long reads, Illumina HiSeq, and Hi‐C data. The estimated genome sizes were 1375.4 Mb (*P. edulis*) and 1357.5 Mb (*P. edulis* f. *flavicarpa*), respectively, with 30,670 and 29,425 protein‐coding genes predicted. Comparative analysis revealed over 8 million SNPs, 1 million insertions/deletions (indels), and ∼142 Mb of presence/absence variations (PAVs). The study also reported a recent whole‐genome duplication (WGD) event and proposed that metabolite differences between the two genotypes may underlie their variation in taste (*P. edulis* f. *flavicarpa*) and aroma (*P. edulis*), offering new leads for metabolic trait improvement (Zheng et al., 2024).

These high‐quality reference genomes provide powerful resources for gene discovery, synteny analysis, and marker development. They also enable the integration of structural variations and pangenomic elements into trait‐mapping pipelines. However, it might be noted that none of these genomes have been haplotype‐resolved.

While reference genomes represent a critical milestone, a single genome cannot capture the full extent of diversity present in landraces, wild relatives, and cultivated varieties. The construction of pangenomes through the sequencing and comparison of multiple diverse genotypes is now recognized as essential for comprehensive trait discovery and crop improvement (Sabety et al., 2024; Tao et al., 2019). In *Passiflora*, such efforts will be instrumental in capturing rare alleles, characterizing structural variations such as PAVs, and enabling more inclusive marker‐assisted and GS strategies.

## 4 FUNCTIONAL OMICS

### 4.1 Comprehensive transcriptomic resources

While genome sequences offer a static blueprint of an organism's genetic potential, functional omics, particularly transcriptomics, provides dynamic insights into gene activity under specific developmental, physiological, or environmental conditions. Genome annotation, a critical step in adding biological meaning to raw sequence data, leverages transcriptomic information to identify coding sequences, untranslated regions, and regulatory elements, thereby facilitating the discovery of functional genes and pathways (Angel et al., 2018). In non‐model and underutilized crops like passion fruit, transcriptome assemblies play a vital role in gene function prediction, trait dissection, and stress response studies. High‐throughput RNA sequencing (RNA‐seq) enables the capture of a wide range of gene expression profiles across different tissues, developmental stages, and environmental conditions (D'Agostino et al., 2022). In recent years, several transcriptomic datasets have been generated for passion fruit, especially in response to abiotic stress.

#### 4.1.1 Response to cold stress

The first *de novo* transcriptome assembly in *P. edulis* was developed from two contrasting cultivars: Pintang 1 (cold‐tolerant) and Purple Fragrance 1 (cold‐susceptible), utilizing the Illumina HiSeq 2500 platform. This study generated 86,880 unigenes with a mean length of 1449 bp (S. Liu et al., 2017). Differential gene expression analysis uncovered key candidate genes and 56 transcription factors potentially involved in cold tolerance. Functional enrichment analysis further implicated these differentially expressed genes (DEGs) in signal transduction, stress response, and cellular homeostasis pathways. Although gene candidates were proposed, the study lacked follow‐up validation or detailed expression profiling, leaving a gap in functional characterization.

In a more recent study, *P. edulis* Huangjinguo (cold‐susceptible) and Tainong 1 (cold‐tolerant) were examined under normal and cold stress conditions (Wu et al., 2021). The transcriptome assembly yielded 47,353 unigenes, and comparative analysis revealed 955 upregulated DEGs under cold stress—a significantly higher number than previously reported. This study provided insights into cold acclimation mechanisms, highlighting the role of protein phosphorylation, signal transduction pathways, and transcriptional regulation in stress adaptation. These findings expanded our understanding of the molecular basis of cold tolerance in *Passiflora*, though functional validation of candidate genes remains limited.

#### 4.1.2 Response to high temperature

High‐temperature stress poses a major challenge to the sustainable cultivation of passion fruit, particularly in tropical and subtropical regions where extreme temperatures can compromise reproductive development, photosynthesis, and fruit set (L. Li et al., 2025; Matsuda & Takaragawa, 2023). Understanding the molecular basis of thermotolerance is therefore critical for breeding climate‐resilient cultivars.

A recent study investigated the heat stress response in a hybrid cultivar of *P. edulis*, Zhuangxiang Mibao, providing a theoretical framework for the transcriptional and physiological mechanisms underlying high‐temperature adaptation (H. Wang et al., 2023). By combining RNA‐seq and biochemical assays, the study identified key regulatory genes and pathways activated under heat stress conditions.

High‐throughput RNA‐seq, using the Illumina NovaSeq 6000 platform, generated 6.89 Gb of high‐quality clean reads. These reads were aligned to the *P. edulis* reference genome reported by Xia et al. (2021), enabling precise identification of 215 DEGs in flower bud tissues exposed to high temperature versus control conditions. The analysis revealed that high‐temperature response in passion fruit is governed by complex transcriptional reprogramming, involving a wide range of genes with divergent expression patterns. Many of these DEGs were linked to pathways regulating hormone signaling, oxidative stress response, heat shock proteins, and transcription factors, suggesting the involvement of a multilayered defence mechanism against heat stress. To better understand the functional significance of these DEGs, physiological measurements were performed on leaf samples under controlled climatic regimes (normal and high temperature). Parameters such as net photosynthetic rate, stomatal conductance, intercellular CO_2_ concentration, transpiration rate, and maximum quantum yield of photosystem II (Fv/Fm) were evaluated. Notably, the physiological indices showed a strong correlation with gene expression profiles, linking transcriptomic shifts to functional phenotypic outcomes. Furthermore, quantitative real‐time PCR (qRT‐PCR) validation of 10 randomly selected DEGs confirmed the reliability of the RNA‐seq results, reinforcing the credibility of the gene expression trends identified. These findings underscore the utility of combining physiological and transcriptomic analyses to uncover candidate genes and pathways associated with thermotolerance in *Passiflora*.

Overall, this integrative approach offers valuable insights for future efforts to breed passion fruit cultivars capable of maintaining productivity under rising temperatures. Functional validation of the identified candidate genes, followed by their deployment through marker‐assisted or GS strategies, could expedite the development of heat‐resilient genotypes.

#### 4.1.3 Response to biotic stresses

Biotic stress, particularly viral and bacterial infections, poses a major threat to passion fruit cultivation, leading to significant losses in yield and fruit quality. These infections pose a significant threat to passion fruit cultivation, as they lead to a continuous decline in fruit yield and quality, often resulting in crop failure and substantial economic losses for farmers, with yield losses as high as 50%–100% been reported in various passion fruit species (Choi, 2025).

Understanding the molecular responses of *Passiflora* species to pathogen attack is essential for developing resistant cultivars and implementing genomics‐assisted disease management strategies. An expression profile of *P. alata* accession SV3 in response to *Xanthomonas axonopodis* pv. *passiflorae* (Xap) infection, the causal agent of bacterial spot disease, was reported in 2023. Sequencing of libraries was performed on Illumina's NextSeq 2000 platform using the paired‐end strategy, and about 300 million raw reads were generated. Using the Benchmarking Universal Single‐Copy Orthologs assessment, a total of 1329 complete genes and 96.6% of the orthologs that were conserved across embryophytes were represented in the assembled transcriptome. However, fragmentation (2.32%) and missing sequences (1.02%) were also reported. A total of 1242 differentially expressed transcripts were identified, and upon further analysis, it was confirmed that *P. alata* is a susceptible host for Xap infection (Cardoso et al., 2023). These findings provide an important foundation for identifying susceptibility genes and guiding efforts to develop bacterial spot‐resistant cultivars in *Passiflora*.

### 4.2 Mechanisms involved in different fruit qualities

Fruit quality in passion fruit is determined by a complex combination of genetic, biochemical, and physiological factors that influence appearance, taste, aroma, and nutritional content. Breeding programs target key agronomic traits such as yield, pulp content, flavor, and fruit appearance, while also addressing post‐harvest physiological challenges that affect shelf life. Understanding heritability and genetic correlations of these traits is essential for optimizing molecular breeding strategies and enhancing productivity and fruit quality in passion fruit cultivars (Sun et al., 2025). As observed in previous studies, positive correlations among traits such as fruit size, weight, diameter, and pulp content support the feasibility of selective breeding to substantially improve yield and fruit quality, while negative correlations—such as those between soluble solids and yield—highlight the importance of balancing trade‐offs in selection strategies, thus guiding breeders on how to achieve a desirable combination of yield and quality (Moraes et al., 2005; G. d. S. Pereira et al., 2017; Sun et al., 2025).

Recent advances in functional genomics, particularly transcriptomics and metabolomics, have been instrumental in dissecting the molecular mechanisms underlying fruit quality traits. High‐throughput sequencing has been integrated with metabolite profiling to explore gene networks involved in epicarp pigmentation, ripening, and aroma development.

#### 4.2.1 Epicarp color

The color of passion fruit peel (epicarp) is an important commercial and consumer trait, varying primarily between purple and yellow cultivars. A combined transcriptome and metabolome analysis from the pulp and peel of two *P. edulis* Sims varieties: Golden Passion Fruit (yellow) and Tainong 1 (purple), led to the discovery of the mechanism of color and fruit formation. The Illumina HiSeq platform was used for sequencing 12 complementary DNA (cDNA) libraries from each fruit, followed by processing of raw reads via Trimmomatic and *de novo* assembly into transcripts using Trinity software. A total of 145,795 unigenes were identified. qRT‐PCR validation of 12 candidate genes playing a role in determining the taste, color, and nutritional quality of the fruit confirmed the RNA‐seq results. Findings from this study revealed that a majority of the evaluated flavonoids, anthocyanins, and flavanols were significantly upregulated in the purple cultivar compared to their levels in the yellow cultivar, suggesting that differential expression of flavonoid biosynthesis genes is a key determinant of fruit color (Qiu et al., 2020).

#### 4.2.2 Fruit ripening

Fruit ripening in passion fruit is a complex process involving hormonal signaling, cell wall modification, and sugar metabolism. Candidate genes involved in ripening and epidermal senescence in *P. edulis*’ variety Tainong1 were identified by exposing various growth stages of the fruit to two postharvest treatments: preservative film and 1‐methylcyclopropene, an ethylene inhibitor. A total of 56,628 unigenes (average length 854 bp) were identified through the Illumina sequencing platform. From this dataset, a total of 9811 DEGs were identified during the ripening stage. Numerous DEGs were identified to be substantially enriched in plant‐hormone signal transduction, starch, and sucrose metabolism, along with phenylpropanoid biosynthesis at the postharvest stage. In accordance with the study's findings, maturation of passion fruit is strongly linked to the pathways regulating hormone signaling and cell wall metabolism (C. Li et al., 2020).

#### 4.2.3 Aroma biosynthesis

Aroma is another key quality attribute, contributing to fruit marketability and consumer preference. The genetic mechanism of aroma synthesis was identified from the purple‐fruited cultivars of *P. edulis*. cDNA libraries were sequenced on Illumina NovaSeq platform with a paired‐end strategy. A total of 376 fruit‐specific genes were identified and found to be significantly enriched in pathways related to flavonoid biosynthesis, anthocyanin‐containing compound biosynthesis, and leucocyanidin oxygenase activity. From this dataset, 45 candidate genes were found to play an important role in the ethylene signaling and aroma synthesis pathways during fruit development, highlighting their potential roles in modulating fruit flavor and aroma (Ma et al., 2021).

Subsequently, a more targeted study using the same cultivar, Tainong1, determined the mechanism of aroma formation during passion fruit ripening. This was the first comprehensive analysis of passion fruit volatile organic compound (VOC) biosynthesis. Different stages of fruit ripening were selected, and 148 VOCs and related DEGs were identified. Illumina sequencing platform was used to create nine cDNA assemblies, which were later assembled *de novo*. A total of 24 unigenes were identified to be enriched in fatty acid metabolism (13 unigenes) and amino acid metabolism (11 unigenes). Combined results from RNA‐Seq data and gas chromatography‐mass spectrometry profiles from this study suggested that nine out of the identified DEGs were involved in alcohol acyltransferase, key enzymes in the biosynthesis of ester compounds, major contributors to the characteristic aroma of passion fruit (J. Li et al., 2021).

Another study exploring pigment biosynthesis reported three key enzyme genes, *chalcone isomerase*, *chalcone synthase*, and flavonoid *3′5′‐h* *ydroxylase* by utilizing an integrated RNA‐seq and metabolome analysis‐based approach. These three genes were identified as key regulators of flavonoid biosynthesis, which not only influence fruit coloration but may also impact aroma and other sensory attributes. Illumina RNA‐seq was used to sequence cDNA libraries, identifying 10,693 DEGs and 295 unigenes involved in various cellular pathways. This study provides insights into the metabolic processes underlying different fruit development stages in passion fruit (Xia et al., 2021).

Gaining insight into gene expression patterns is crucial for understanding how the underlying genome sequence regulates specific plant phenotypes at key developmental stages of the plant. The development of a comprehensive gene expression atlas for different genotypes of passion fruit using RNA‐seq data from different organs and plant developmental stages would be very beneficial. Such atlases have been successfully developed in other horticultural crops and have accelerated functional genomics research and crop improvement (Roy et al., 2024; Sánchez‐Sevilla et al., 2017; Xanthopoulou et al., 2021).

### 4.3 Integrated omics for a comprehensive understanding of plant biology

Despite substantial progress in genomic and transcriptomic research, the genetic regulation of complex traits such as flavor, aroma, and nutritional quality in *Passiflora* remains only partially understood. A major limitation of current studies is their predominant focus on postharvest stages, which restricts insights into the dynamic molecular and metabolic processes that occur throughout fruit development. Furthermore, metabolite profiling in passion fruit has typically been confined to a limited subset of compounds, potentially missing key volatile and non‐volatile metabolites that contribute to taste and aroma.

To overcome these limitations, the integration of multi‐omics approaches, including transcriptomics, proteomics, metabolomics, epigenomics, and metabolic flux analysis, is essential. When combined with high‐resolution phenotyping across multiple developmental stages, these approaches provide a powerful systems‐level framework to dissect the molecular basis of agronomically and nutritionally important traits. Such integration will be pivotal in identifying robust candidate genes, regulatory networks, and molecular markers for use in quality‐focused breeding programs.

Multi‐omics strategies help bridge the genome‐to‐phenome gap by associating genotype‐derived data with downstream biological outcomes (Choi, 2019; Langridge & Fleury, 2011). By correlating transcript, protein, and metabolite abundance with phenotypic trait variation, these platforms enable the discovery of causal mechanisms and biomarkers that cannot be identified through genomic data alone. In particular, integrated omics enhances positional cloning by linking target loci with specific molecular changes, such as mRNA abundance shifts or protein accumulation patterns associated with key phenotypes (Taagen et al., 2021; Y. Wang et al., 2020).

For instance, a comparative proteomic analysis of four different tissues from the fruit of *P. edulis* identified 295 differentially expressed proteins associated with lipid peroxidation, oxygen scavenging, response to heat stress, and pathogen resistance. These findings provided novel insights into regulatory and stress‐responsive networks in passion fruit during storage (Garcia et al., 2024). Moreover, a ^1^H nuclear magnetic resonance spectroscopy‐based metabolomics approach identified 30 key metabolites that were differentially regulated during the maturation and ripening of passion fruit (Md Nor et al., 2022). Furthermore, two recent integrative functional omics studies revealed the regulatory mechanisms of flavonoid biosynthesis in the skin of *Passiflora* sp. (Fang et al., 2025) and anthocyanin biosynthesis regulations in the pericarp of *P. edulis* (Chen et al., 2025). The former study identified 151 flavonoid metabolites, including 25 key metabolites potentially linked to the purple phenotype, and proposed a key candidate gene (*PeMYB114*) for molecular breeding to improve peel color traits in passion fruit. The latter study identified a total of nine differentially expressed proteins involved in the flavonoid metabolic process in the pericarp of *P. edulis*, laying a foundation for the subsequent exploration of the regulatory mechanism of anthocyanin biosynthesis and the functional identification of related genes.

### 4.4 Genome editing for precise and rapid trait improvement

Another approach for the precise and targeted trait enhancement in passion fruit is genetic engineering. This approach offers a promising avenue for improving the cultivation of passion fruit varieties with improved resistance against biotic and abiotic stresses and enhanced fruit quality production by facilitating the accurate introduction of genetic variations, thus replicating the genetic diversity observed in wild relatives or closely related species. These targeted modifications of specific genes or regulatory elements could also unlock untapped genetic potential and expand the available variation for crop enhancement, thereby fostering the development of resilient, productive, and nutritionally valuable crops, as was observed in the case of tomato (*Solanum lycopersicum*), rice (*Oryza sativa*), maize (*Zea mays*), soy (*Glycine max*), carrot (*Daucus carota*), barley (*Hordeum vulgare*), potato (*Solanum tuberosum*), and wheat (*Triticum aestivum*) (Daniel et al., 2023; Ghogare et al., 2020; Nekrasov et al., 2017; Ortigosa et al., 2019; M. Wang et al., 2017; W. Wang et al., 2019). In this regard, recent advances in passion fruit include the development of an efficient *Agrobacterium*‐mediated *in planta* transformation system with a reported regeneration efficiency of 86% and transformation efficiency of 29%. Successful transformation was confirmed at the DNA and RNA levels and by β‐glucuronidase (GUS) staining and green fluorescent protein measurements (Rizwan et al., 2021). In another study, a viable in vitro transformation protocol for Uganda's yellow passion fruit, directly from leaf discs, was developed using *GUS* reporter gene, and a transformation efficiency of 0.456% was observed (Tuhaise et al., 2019). These protocols will contribute to the genetic improvement of passion fruit breeding. Various tissue culture techniques have also been applied to different *Passiflora* species, enabling both indirect and direct shoot regeneration from different explant types (Lima et al., 2000). Genetic engineering approaches aim to enhance disease and pest resistance, as well as other agriculturally relevant traits. Furthermore, the application of this alternative approach for passion fruit improvement could benefit from the application of clustered regularly interspaced short palindromic repeats (CRISPR) and associated proteins (CRISPR/Cas)‐based gene editing systems, as has been observed in many fruits, ornamental, and industrial crops. Most horticultural crops are recalcitrant to in vitro manipulation; therefore, as advances are made in the regeneration of such crops, targeted gene editing via CRISPR/Cas9 will enable rapid trait improvement, overcoming long breeding cycles. As research progresses, the application of targeted editing of genes that regulate desirable traits related to nutrition, novel organoleptic properties, and novel industrial uses can help develop better passion fruit varieties, thus playing a significant role in passion fruit breeding and improvement programs.

## 5 TOWARD SEQUENCE‐BASED BREEDING

The genus *Passiflora* comprises many genetically diverse species, most of which face significant biotic and abiotic challenges that limit the development of new cultivars. These challenges include susceptibility to diseases like dieback, fusarium wilt, and woodiness virus (Table 4), and various abiotic stresses such as fluctuating temperature, drought, and high salinity (Table 5). Breeding programs, with the aim of improving passion fruit varieties for horticultural, ornamental, medical, and functional food purposes, must address these issues to ensure compatibility across all uses (Cerqueira‐Silva et al., 2018).

**TABLE 4:** Biotic stresses impacting passion fruit production.

Type	Name	Causal agent (s)	Effects observed in passion fruit	References
Diseases	Cucumber mosaic disease	Cucumber mosaic virus	Bright yellow mottling on leaves starting at random points on the vine and later diminishing in intensity toward the tip	Fischer and Rezende (2008)
	*Passiflora* latent infection	Passiflora latent virus	Inconspicuous, systemic foliar mosaic; older leaves are mottled in cooler weather	Bao et al. (2023)
	Passion fruit yellow mosaic infection	Passion fruit yellow mosaic virus	Characteristic bright yellow mosaic, yellow net, and leaf crinkle	Morales et al. (2002)
	Passion fruit woodiness disease	Passion fruit woodiness virus	Mosaic‐like symptoms observed on various plant parts, rugose leaves with chlorotic and/or ring spots, distorted fruit, woody pericarp, reduced vigor, and lifespan	Elliot (2015)
	Passion fruit vein‐clearing disease	Passion fruit vein‐clearing virus	Vein clearing symptom on the leaves, reduced size of leaves and fruit	Fischer and Rezende (2008)
	Fusarium wilt	*Fusarium oxysporum*	Reddish‐brown discoloration of water‐conducting tissues of the stem and root, stunted growth, yellow leaves with premature falling, plant wilts and dies	Rooney‐Latham et al. (2011)
	Collar rot	*Phytophthora parasitica* and *Phytophthora cinnamomi*	Bark splitting at the plant base, whole vine wilts, turns yellow, and eventually dies	Young (1970)
	Root and crown rot	*Phytophthora nicotianae* and *Phytophthora cinnamomi*	Purpling and reddening of older leaves, darkened bark and wood tissue, crown rot canker, plants wilt and die rapidly, oozing of gum or dark sap, fruit rot, and root decay	Burgess et al. (2021)
	Anthracnose/septoria/alternata/brown/sunken/gray spots	*Colletotrichum gloeosporioides*, *Septoria passiflorae*, *Alternaria alternata*, *Alternaria passiflorae*	Dark and sunken spots or lesions on affected foliage, stems, and fruit; post‐harvest losses; small black spots on affected areas that turn parchment‐like; and the skin easily breaks	Fischer and Rezende (2008)
	Stem Bulging	*Gibberella fujikuroi* and *Fusarium* sp.	Lethal condition, poor plant growth, bulged main and lateral branches, blocked vascular system	Wanniarachchi et al. (2017)
Pests	Fruit fly infestations	Queensland fruit fly (*Bactrocera tryoni*), Mediterranean fruit fly (*Ceratitis capitata*)	Rots, discoloration of fruit, woody lumps on skin, and fruit drop	Rojas‐Sandoval et al. (2014)
	Fruit spotting bugs (FSB)	*Amblypelta lutescens lutescens*, *Amblypelta nitida*	Brown lesions on the seed and small black “pin pricks” on the internal white surface of the skin, wilting shoots, and 50% yield losses have been reported	The Passion Vine (2019)
	Red scale	*Coccus hesperidum* Linnaeus, *Aonidiella aurantia*	Leaf drop and dieback	Business Queensland (2022)
	Mealybugs infestation	*Hypogeococcus pungens*	Hindered plant growth, leaf drop, sap sucking	Business Queensland (2022)

**TABLE 5:** Abiotic stresses impacting passion fruit production.

Type	Effects observed in plants	Reference
Drought	Reduced flowering and fruit set, small fruits with low pulp content, premature fruit drop, and increased susceptibility of seedlings to Fusarium wilt	Kondo and Morizono (2022); Lozano‐Montaña et al. (2021)
Chilling injury	Fruit skin discoloration (can penetrate skin or exocarp), pitting, water‐soaked areas, uneven ripening, increased decay, severe debilitation of vines, increased susceptibility to blind tipping	Liu et al. (2017); Wu et al. (2021)
Salinity	Pale and thin‐skinned fruits, more acidic juice, and increased chances of shriveling	Rigden (2011)
High temperature stress	Restricted growth, short flowering period, few flower buds, low fruit setting rate, severe fruit drop, and more deformed fruit	Matsuda and Higuchi (2020)

Genomics‐assisted breeding offers a promising solution, as demonstrated in crops like wheat and barley, where genetic tools have been used to combat both biotic and abiotic stresses (Riaz et al., 2021; Varshney et al., 2007). In passion fruit, emerging genomic studies have identified key resistance genes, such as *CC‐NBS‐LRR*, which are involved in pathogen resistance and stress response (Zia et al., 2024). Additionally, genomic tools like genome‐wide association studies (GWAS) and MAS hold potential to identify and select for resistance traits, hence streamlining the breeding process.

**FIGURE 2:**
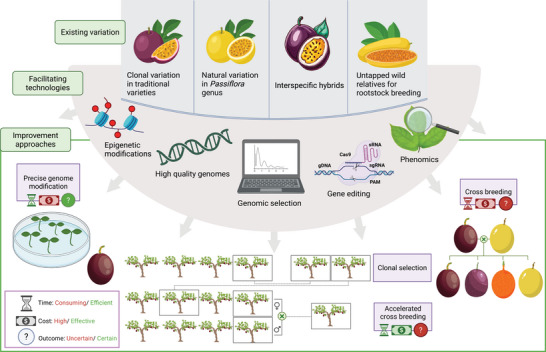
Evolution of passion fruit breeding strategies and future potential. This figure illustrates the transition from traditional passion fruit breeding approaches, characterized by phenotypic selection and hybridization leading to prolonged breeding cycles, to the envisioned integration of modern tools. The successful incorporation of genomics, epigenomics, advanced biotechnology, and predictive breeding methodologies is critical for significantly enhancing breeding efficiency and accelerating genetic gains in passion fruit.

These genomic insights, along with advanced phenotyping and modern breeding techniques, offer a strategic approach to enhance passion fruit resilience and productivity in challenging environments. Moreover, integrating multi‐omics approaches such as genomics, transcriptomics, proteomics, and metabolomics will deepen our understanding of plant responses at different molecular levels. This systems biology perspective enables the identification of robust molecular markers, regulatory pathways, and candidate genes for targeted trait improvement, advancing the development of resilient passion fruit varieties suitable for a wide range of environments (Figure 2). However, several barriers must be overcome before the full potential of sequence‐based breeding in *Passiflora* can be realized. Significant among these are the limited genetic diversity within cultivated germplasm and the complex reproductive systems in *Passiflora* species. To address these challenges, breeders can harness genetic variation from wild relatives through introgression breeding, somaclonal variation, and modern technologies like gene editing. The development of efficient transformation and regeneration systems is also crucial for enabling gene editing and functional validation of key loci. In addition, strategies like collaborative breeding programs, genetic resource conservation, and hybrid breeding can further expand the genetic base available in passion fruit selection. A comprehensive analytical framework incorporating both ecological and economic considerations into breeding frameworks will also be essential for optimizing cultivar performance across diverse agroecological zones. Together, these strategies will support the transition toward sequence‐based breeding and contribute to the development of high‐performing, resilient passion fruit cultivars tailored to future climatic and market demands.

## 6 DATA REPOSITORIES TO PROVIDE CENTRALIZED ACCESS TO EXTENSIVE DATA

The advent of high‐throughput genomic sequencing technologies has led to the generation of vast omics data for passion fruit, including organellar genomic resources, high‐quality reference genome assemblies, transcriptomic datasets (RNA‐seq), DNA markers, genetic maps, and QTLs. While these resources have greatly expanded our understanding of passion fruit biology, their full utility depends on centralized, well‐structured, and user‐friendly data repositories. The ability to integrate, visualize, and analyze these large datasets is essential for facilitating data‐driven research and accelerating breeding efforts.

To address this need, the passion fruit genomic database (PGD) was recently developed to store, analyze, and integrate large‐scale datasets, enabling researchers to leverage omics data for passion fruit improvement (Yu et al., 2024). PGD is a rich resource for passion fruit research, featuring a wide array of omics data, including genome sequences, gene structures, and expression profiles. Its website provides various interactive analysis tools, enabling users to perform tasks like basic and batch queries, download options, a genome browser, sequence similarity searches through a basic local alignment search tool, screening and analysis of gene families, gene structure analysis, determination of the physicochemical properties of proteins, conserved protein motif analysis, gene ontology enrichment analysis, and Kyoto encyclopedia of genes and genomes pathway, as well as calculation of the substitution ratios (Ka/Ks) of orthologous gene pairs.

## 7 WAY FORWARD FOR PASSION FRUIT IMPROVEMENT

With growing challenges posed by climate change, emerging pathogens, and increasing market demands, modern breeding of passion fruit must adopt integrative and cutting‐edge genomic strategies. While considerable progress has been made in generating reference genomes, transcriptomes, and genetic markers, there remains a need to leverage these tools through advanced breeding approaches such as telomere‐to‐telomere (T2T) genome assemblies, pangenomics, GWAS, haplotype‐based breeding, and GS. Together, these cutting‐edge approaches will accelerate the development of climate‐resilient, high‐quality, and high‐yielding passion fruit cultivars.

### 7.1 Toward developing telomere‐to‐telomere genome assembly for passion fruit

Advancements in sequencing methodologies, notably long‐read sequencing, have facilitated the development of gapless T2T genome assemblies, which are instrumental in realizing the complete potential of crop translational genomics and offer a new and comprehensive understanding of genome organization and function. Genomic events in plants, like the continuum of polyploidy from ancient species to modern cultivars, as well as highly similar and long repetitive elements, pose a great difficulty for the assembly and analysis of plant genomes. In the past, passion fruit has undergone a core eudicot‐common whole‐genome triplication event and a more recent WGD event, resulting in difficulties in the genome analysis of various species of *Passiflora* (Ma et al., 2021). The development of a haplotype‐resolved, gapless T2T assembly in passion fruit will help elucidate the roles of repetitive elements in gene regulation and in pangenomics, functional genomics, genome‐assisted breeding, and targeted genome manipulation. In conjunction with sequence‐enriched germplasm repositories and multi‐omics integration, T2T assemblies thus hold great promise for basic and applied plant sciences.

### 7.2 Pangenome and super‐pangenome for comprehensive insights into genetic diversity

Reference genomes, combined with whole‐genome re‐sequencing data, have immense potential to support crop breeding efforts by providing insights into the available genetic diversity within a species. However, despite advancements in sequencing technologies that have enabled the development of high‐quality reference genome assemblies, a single reference genome remains insufficient to fully represent a species, as it cannot encapsulate all the genetic diversity present across different cultivars, even within the same species (Khan et al., 2020). One major limitation of relying on a single reference genome is that it does not account for the genetic diversification and structural alterations that occur due to long‐term domestication. As a result, important genetic variations may be overlooked, as has been observed in crops such as cotton (*Gossypium herbaceum*), tomato, soybean, and Asian cultivated rice (*Oryza sativa*) (Gao et al., 2019; J. Li et al., 2021; Y. Liu et al., 2020; W. Wang et al., 2018). Variants linked to specific phenotypes may go unnoticed when using a single genome as a reference. To overcome these limitations, pangenome approaches are increasingly being deployed to capture the full genetic diversity within a species. The concept and applications of pangenome, including core (conserved across all individuals) and dispensable (variable among individuals) genomes, have been well described by Hurgobin and Edwards (2017). These resources are particularly valuable for crops like passion fruit, which suffer from a narrow genetic base among cultivated accessions and present a major bottleneck to genetic improvement. Core genes are generally relatively conserved across species, as they perform critical functions, while dispensable genes exhibit a high level of variability among different species, thereby helping them adapt to various environmental conditions (Danilevicz et al., 2020; Sun et al., 2020; Tao et al., 2019). Thus, breeding efforts for passion fruit should focus on the dispensable genome to incorporate variations that influence responses to biotic and abiotic stresses, thereby enhancing crop improvement programs. Moreover, pangenomes constructed from diverse individuals are gaining popularity as a tool for identifying precise genetic variations, providing a more comprehensive view of a species’ genetic diversity. This is particularly relevant for large structural variants (SVs) such as PAVs, copy number variants, translocations, and inversions. These SVs play a crucial role in phenotypic diversity, adaptation, and selection history (Raza et al., 2023). Since SVs are inheritable, they can significantly contribute to future breeding strategies aimed at improving crop efficiency and developing climate‐smart crop varieties. Furthermore, shifting from a single genome to a pangenome‐based approach will enhance variant calling and facilitate the identification of genes associated with key agronomic traits, ultimately advancing crop breeding and genetic research (Golicz et al., 2020).

A comparison of wild accessions representing different gene pools reveals copy number variations and presence‐absence mutations that are predicted to be associated with positive selection and agronomically important traits. To capture this broader genetic diversity, a “super‐pangenome” approach has been proposed, which involves constructing a pangenome from the pangenomes of multiple species within a given genus (Khan et al., 2020). For *Passiflora* species, *de novo* genome assemblies are being developed for various species, and there have been some efforts to resequence selected accessions. This information may be leveraged in the future to construct a *Passiflora* super‐pangenome. A super‐pangenome would enable the discovery of conserved and divergent genomic elements across species, allow the comparative analysis of gene family expansions, contractions, and diversification, improve gene annotation and identification of lineage‐specific adaptations, and give broader insights into domestication, speciation, and trait evolution within the genus. The development of both a passion fruit pangenome and a super‐pangenome would provide a powerful resource for bridging the genome‐to‐phenome gap and would facilitate the discovery and utilization of superior alleles associated with yield, quality, stress tolerance, and novel market traits, laying a comprehensive genomic foundation for future breeding and biotechnology efforts in *Passiflora* improvement.

### 7.3 Genome‐wide association studies for deciphering agronomically important traits

GWAS is a powerful statistical genetic method that offers higher‐resolution discovery of genes controlling crucial agronomic traits. GWAS leverages natural genetic variation across diverse germplasm to discover marker‐trait associations, offering deeper insights into the genetic architecture of traits such as yield enhancement to the biosynthesis pathways of bioactive compounds (Alseekh et al., 2021). Some notable recent examples include deciphering the genetic mechanism of fruit quality in *Punica granatum* (pomegranate) the identification of markers associated with biotic stress response and genotype‐by‐environment interactions in *Prunus persica* (peach) and *Prunus armeniaca* (apricot) (Chen et al., 2025; Zahid et al., 2022). At present, GWAS applications in passion fruit remain limited. The deployment of GWAS to dissect complex traits will allow for the identification of DNA markers associated with critical agronomic traits to improve the productivity of passion fruit. Furthermore, through large‐scale genotyping and phenotyping of diverse accessions, GWAS can reveal SNPs and QTLs associated with complex traits such as disease resistance (e.g., woodiness virus or fusarium wilt), fruit quality attributes (e.g., increased sugar content, aroma), and productivity (e.g., yield, fruit size, shelf life). These identified genomic regions, often harboring desirable alleles from wild relatives or landraces, can then be efficiently introgressed into elite commercial passion fruit lines through marker‐assisted backcrossing, a core strategy within genomics‐assisted breeding. The development of highly specific and cost‐effective SNP‐based markers, such as those utilized in Kompetitive allele‐specific PCR marker assays, will revolutionize passion fruit genotyping by enabling high‐throughput analysis on low‐cost genotyping platforms. This integrated approach will facilitate the precise and rapid introgression of agronomically important traits like disease resistance, improved fruit size, or enhanced shelf life into recipient passion fruit lines, significantly accelerating the development of improved, resilient, and market‐desirable passion fruit varieties.

An emerging and complementary strategy that can enable the “designing” of future crops with desired traits while demanding less monetary investment, and in the absence of challenging public acceptance, is haplotype‐based breeding. This strategy promises assembly of the desired haplotype combinations in elite crop varieties and subsequently has the potential to enable informed breeding decisions (Bhat et al., 2021). For instance, a combination of superior haplotypes of previously validated genes that confer increased sugar content, higher aril‐to‐seed ratio, larger fruit size, reduced endocarp thickness, and higher yield can be used to design crop ideotypes for improved flavor and aroma.

### 7.4 GS to accelerate crop genetic improvement

The application of MAS has revolutionized conventional crop improvement systems. Techniques like QTL mapping help identify major‐effect loci governing agronomically important traits. However, many of these agronomically important traits are complex and influenced by numerous minor‐effect loci, which MAS often fails to capture (Desta & Ortiz, 2014). To address this limitation, GS was proposed by Meuwissen et al. (2001) as a more sophisticated breeding strategy. GS utilizes high‐throughput genotyping technologies to analyze genetic markers across the genome. Two populations are used: a “training population” comprising individuals with desirable traits and a “breeding population/testing population.” The individuals in the training population are genotyped and phenotyped to train the prediction model, and thus, this population informs the model about the relationships between genetic markers and traits. Furthermore, the gathered information is applied to the already genotyped individuals from the “breeding population” to predict the same traits. The elimination of phenotyping and an in‐depth analysis of individual loci in the breeding population significantly accelerates the process of creating new cultivars with desirable traits, as has been observed previously in wheat, cassava (*Manihot esculenta*), and soybean (Ahmar et al., 2020; Atanda et al., 2021; Bose et al., 2024; Oliveira et al., 2012; Sinha et al., 2023). However, an effective implementation of modern quantitative genetic approaches, including GS, requires large‐scale field trials across multiple environments to accurately estimate genetic parameters. Furthermore, there is a need to assess the training population at an adequate developmental age within each environmental stratum. This thorough phenotyping is essential because genotype × environment (G × E) interaction severely compromises the transferability and accuracy of predictive models across different regions (Oliveira et al., 2012).

Recently, the potential of GS has been successfully integrated into horticultural breeding programs to enhance trait prediction. In crops like capsicum (*Capsicum annuum*), apple (*Malus domestica*), grapevine (*Vitis vinifera*), and tomato. This approach has been applied to predict phenotypic traits like fruit dimensions (length, width, shape, and weight), fruit texture and firmness, and to accelerate the development of varieties with improved agroeconomic traits and to improve fruit quality and resistance to bacterial wilt, respectively (Brault et al., 2024; Hong et al., 2020; Roth et al., 2020; Yeon et al., 2024).

On the other hand, the substantial volume of data being generated by these models, combined with the complex relationships therein, impedes a comprehensive understanding of the complex mechanisms behind genes driving the agronomic trait formations. Consequently, the development of a systematic framework to organize this information is necessary, and even though much of this information is currently accessible, it frequently exists in a disaggregated state. This proposed system aims to transform raw data into actionable breeding decisions and thus only considers those tools that are useful to facilitate decision‐making processes in crop breeding. Furthermore, in the current breeding methods, linking genotype to phenotype remains a massive challenge and impedes the optimal application of high‐throughput field phenotyping, genomics, and enviromics. Artificial intelligence (AI) has emerged as a promising tool for managing high‐dimensional genotypic data complexity (Guo et al., 2023). Leveraging AI to analyze complex omics datasets enables rapid gene identification, a critical step in accelerating crop improvement programs. Specifically, this approach facilitates precision breeding for passion fruit, allowing for the selection and development of cultivars with enhanced adaptation to challenging environmental conditions, ultimately boosting production.

## 8 CONCLUSIONS AND FUTURE PERSPECTIVES

Despite its nutritional and economic value, passion fruit remains an underutilized crop with limited breeding progress. Improving breeding efficiency is essential to enhance productivity, fruit quality, and resilience to biotic and abiotic stresses. At the heart of this improvement lies the effective harnessing of genetic variation, both by expanding the genetic base and by accelerating the identification and selection of superior alleles for key agronomic traits. Recent advances in sequencing, high‐throughput phenotyping, and integrative omics have transformed the way breeders approach crop improvement. In passion fruit, genomic resources such as reference genomes, genetic markers, transcriptomes, and expression profiles now offer a strong foundation for unlocking trait architecture and enabling precision breeding. The application of these tools can substantially accelerate breeding cycles, improve selection accuracy, and deliver market‐preferred varieties tailored to consumer and environmental demands. Genomics‐assisted breeding, supported by robust databases and bioinformatics pipelines, is particularly valuable for tackling complex challenges such as disease susceptibility (e.g., woodiness virus, fusarium wilt), loss of vigor, blind tipping, and environmental stress tolerance. Functional genomics, coupled with genome‐wide selection and haplotype‐based breeding, will further enhance the discovery and deployment of candidate genes and elite alleles for traits related to yield, quality, and adaptation. Looking ahead, the implementation of T2T genome assemblies, pangenomics frameworks, genome editing (e.g., CRISPR/Cas), and AI‐based breeding platforms holds transformative potential. These innovations will enable the development of climate‐smart, resilient, and nutritionally enhanced cultivars, reducing breeding timeframes and bridging the genotype‐to‐phenotype gap more effectively than ever before.

In conclusion, the integration of modern genomic technologies into passion fruit improvement programs represents a timely and powerful opportunity to modernize breeding pipelines. Delivering the next generation of high‐yielding, disease‐resistant, and climate‐resilient passion fruit varieties will have far‐reaching impacts, enhancing farmer livelihoods, diversifying horticultural systems, and contributing to global food and nutritional security.

## AUTHOR CONTRIBUTIONS

**Khushboo Fulara**: Writing—original draft; writing—review and editing. **Vanika Garg**: Writing—original draft; writing—review and editing. **Xinhang Sun**: Writing—review and editing. **Rebecca Ford**: Writing—review and editing. **Natalie Dillon**: Writing—review and editing. **Bruce Topp**: Writing—review and editing. **Robert J Henry**: Writing—review and editing. **Mobashwer Alam**: Writing—review and editing. **Rajeev K Varshney**: Conceptualization; writing—review and editing.

## CONFLICT OF INTEREST STATEMENT

The authors declare no conflicts of interest.

## Data Availability

There are no original data associated with this article. Referenced data are available in the literature.
